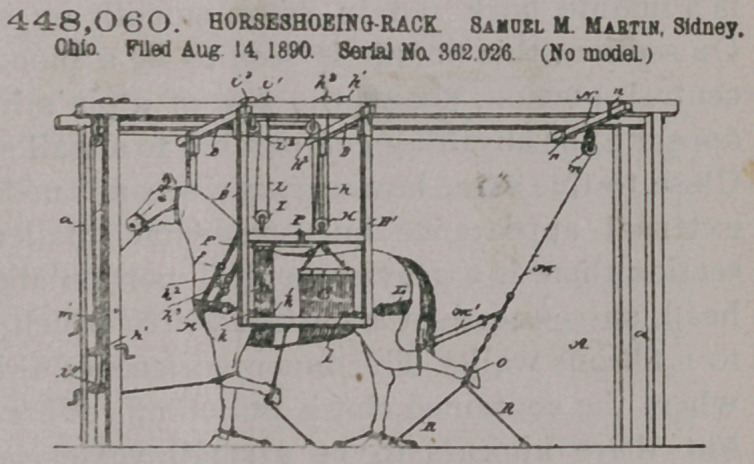# Recent Patents

**Published:** 1891-04

**Authors:** 


### RECENT PATENTS

RELATING TO

### VETERINARY MEDICINE AND ANIMAL INDUSTRY.

*Issued by U. S. Patent Office for Month ending March 14th, 1891.*

**447,466. HAME.** Ralph E. Krumm, Vicksburg, Mich. Filed May 22, 1890. Serial No. 352,695. (No model.)

*Claim.*—1. An adjustable hame consisting of the two parts, one sliding within the other, the outer part being provided with the notches and with the slot leading into its hollow interior, and the inner part being provided with the pivoted latch and being also provided with the rein-ring eye, substantially as set forth.

2. An ajustable hame comprising the two parts, one sliding within the other, the outer part being provided with the notches and the slot leading into its hollow interior and with the pin-hole also leading into its hollow interior, and the inner part being provided with the latch attached thereto by a detachable pivot, said latch being extended out through a slot in the outer part of the hame and turned at an angle to engage the notches, and said inner part being also provided with the rein-ring eye attached thereto by a detachable pin, substantially as set forth.

**447,416. HORSESHOE. WILLIAM SOMERVILLE, Sr., Buffalo, N. Y. Filed Dec. 12, 1890. Serial No. 374,435. (No model.)**

*Claim.*—1. The combination, with a horseshoe provided with a raised tow-plate B, having a flat bearing-face *b* below the body of the shoe, with projecting ribs *b*1 formed on said face and separated by a central space *b*2, and heel-plates C, having flat bearing-faces *c* and projecting ribs *c*1, of a removable toe-calk D, provided with a flat bearing-face *d*, having flanges D1, adapted to rest against the bearing-face of the toe-plate between the projecting ribs of said plate, removable heel-calks J, provided with flat bearing-faces having lateral flanges and adapted to rest against the bearing-faces of the heel-plates between the projecting ribs of said plates, vertical studs or pins formed on both the toe and heel calks and engaging in sockets formed in the plates, and fastening-screws whereby the removable calks are secured to the raised plates, substantially as set forth.

2. The combination, with a horseshoe provided with a raised toe-plate B, having a flat bearing-face *b* below the body of the shoe, with projecting ribs *b*1 formed on said face and vertical ribs *b*2 formed on opposite ends of said toe-plate, of a removable toe-calk D, provided with a flat bearing-face *d*, having lateral flanges *d*1 and adapted to rest against the bearing-face of the toe-plate between the projecting ribs *b*1 of said plate, and vertical lips *h*, formed on opposite ends of said toe-calk and adapted to fit between the vertical ribs *b*3, formed on the ends of the toe-plate, and fastening-screws *i*, engaging in openings formed in the lips *h* and end walls of the toe-plate, substantially as set forth.

**447,611. TOOL FOR DEHORNING CALVES. CHARLES T. INGRAHAM, Dwight, assignor of one-half to Samuel W. Gammon, Chicago, Ill. Filed June 19, 1890. Serial No. 355,962. (No model.)**

*Claim.*—1. In a dehorning-tool, the combination, with two knife-arms pivoted together at the top, of knives secured to the free ends of the said arms and having V-shaped cutting-edges, the V-shaped cutting-edges being reversely formed, substantially as shown and described.

2. In a dehorning-tool, the combination, with two knife-arms pivoted together at the top, of knives secured to the free ends of the said arms and having V-shaped cutting-edges, and stops formed on the said knife-arms to limit the closing movement of the said knives, substantially as shown and described.

3. In a dehorning-tool, the combination, with two knife-arms pivoted together at the top, of knives secured to the free ends of the said arms and having V-shaped cutting edges, and a handle secured on one of the knife-arms and standing at or about at right angles to the same, substantially as shown and described.

4. In a dehorning-tool, the combination, with two knife-arms pivoted together at the top, of knives secured to the free ends of the said arms and having V-shaped cutting edges, a handle secured on one of the knife-arms and standing at or abou-at right angles to the same, and stops formed on the inner edges of the said knifet arms and arranged opposite each other, so as to limit the closing movement of the said knives, substantially as shown and described.

5. In a dehorning-tool, two oppositely-arranged dished knives having V-shaped cutting-edges, the V-shaped cutting-edges being reversely formed, substantially as shown and described.

**447,649. HORSE-COLLAR.** JOHN F. TRAUTMANN, St. Louis, Mo. Filed Oct. 27, 1890. Serial No. 369,486. (No model.)

*Claim.*—1. In a horse-collar, the combination of the roll, facing and backing, the outer edge of the facing being connected to the backing and the outer edge of the backing being folded over the point of connection between it and the facing and being itself secured to the facing at a point remote from the edge of the latter, substantially as set forth.

2. In a horse-collar, the combination, with the roll, facing and backing, of a narrow strip secured to the under or inner side of the backing and to which strip the outer edge of the facing is connected, the outer edge of the backing being folded over the point of connection between said strip and facing and being itself secured to the facing at a point remote from the edge of the latter, substantially as set forth.

**446,828. HOOF-EXPANDER.** GEORGE T. CHAPMAN, White Plains, assignor of one-half to Wm. Harvey Merritt, New York, N. Y. Filed Mar. 19, 1890. Serial No. 344,454. (No model.)

*Claim.*—The combination, with the bar having the prong *b* at one end, the slot in the opposite end portion, and the screw-tapped lug on one side of the movable prong having the notches for receiving the members of the slotted bar, and the notch in the end opposite the prong for the adjusting-screw, also the adjusting-screw, said screw having the collars embracing the sides of the moving prong, all substantially as described.

**446,471. HOOF-EXPANDER AND FROG-DEVELOPER FOR HORSES.** DAVID ROBERGE, Jr., New York, N. Y. Filed July 2, 1889. Serial No. 316.327. (No model.)

*Claim.*—1. The combination, with the spring hoof-expander, of a frog developer and protector connected with and receiving its support from the hoof-expander, substantially as set forth.

2. The combination, with the spring hoof-expander, of a frog-protector, connected at one end to the spring hoof-expander, and adapted to extend below the frog of the horse's hoof, substantially as set forth.

3. A spring hoof-expander and frog-developer formed of metal, the frog-developer being united with the coils at the ends of the springs forming the hoof-expander, substantially as set forth.

**447,793. HORSE-BLANKET.** ALBERT F. RANSOM, Burlington, Wis. Filed Nov. 29, 1890. Serial No. 373,007. (No model.)

*Claim.*—1. The combination, with a horse blanket or cover provided with the bands or stays *a* of fabric, of the fabric breast-stays *b*, one having a snap-hook and the other a ring to be engaged by said hook, said ring and hook being each provided with a cross-bar to engage the said blanket or cover, and thus divide the strain between the latter and the said stays.

2. A horse blanket or cover provided at the upper side of its neck portion with a semi-rigid stiffening-frame *e*, stitched inside of the cover fabric and arranged to straddle the withers and hold the blanket or cover in place and prevent it from sawing back and forth, and thus wearing the mane.

3. A horse blanket or cover provided at the upper side of its neck portion with a semi-rigid stiffening-frame *e*, secured inside of the cover fabric and arranged to straddle the withers and hold the front end of the cover in place, the said cover having at its rear end an imperforate or non-open hood A1, of suitable length to extend down over the upper part of the animal's tail, and the said cover being open below the said hood portion to permit the animal to use his tail freely, as set forth.

**48,092. IMPLEMENT FOR DEHORNING CATTLE.** GEORGE H. STOUT, Marceline, Mo. Filed Nov. 29, 1890. Serial No. 373,059. (No model.)

*Claim.*—1. The combination, in an implement for dehorning cattle, of the plates B B, rigidly secured to a handle so as to be on a line therewith, said plates being connected to each other and provided with correspondingly-shaped openings B1, located one above the other, so as to provide bearings above and below the cutter, a movable cutter having an inclined cutting-edge located between said plates and provided on one side with a rack-bar, and a lever pivoted between the plates and provided with a toothed portion which engages with the rack-bar, whereby the implement can be rigidly held while the cutter is being operated, substantially as shown, and for the purpose set forth.

2. In a dehorning implement, the combination of the handle A, plates B B, located one above the other and provided with oval apertures, said plates being rigidly secured to each other and to the handle, a strip *b*, terminating so as to provide an opening through which the sliding cutter may be passed and a straight portion against which said sliding cutter bears, and a lever removably secured to the side pieces for operating the sliding cutter, substantially as shown, and for the purpose set forth.

**447,919. CATTLE OR STOCK CAR.** Davis L. Sylvester, Philadelphia, Pa., assignor to the Allison Manufacturing Company, of Pennsylvania. Filed May 2, 1889. Serial No. 309,341. (No model.)

*Claim.*—1. A cattle or stock car having an additional floor composed of separate parts independent of the floor proper, bridges having ends adapted to rest on the said floors, mechanism for operating the floors, substantially as described, and sliding guards adapted to work in guides on the sides of the car and be projected longitudinally beyond the end of the car on each side of the bridges, and end blinds, said parts being combined substantially as described.

2. In a cattle-car, the combination of the blinds consisting of slats connected by the links or chains L and having ears at their ends, the guiding-rods G, and the chains H, connected to the lowest of the slats and to the drum J, mounted on the shaft K, said shaft being jouralled in the car-body, substantially as described.

3. In a cattle or stock car having bridges in connection with the lower floor thereof, the sliding guards R, adapted to work in guides on the sides of the car and be projected longitudinally beyond the end of the car and on each side of the said bridges, substantially as described.

4. A cattle or stock car comprising a floor proper, a movable floor made in separate parts, bridges in connection with said floors, sliding guards at the ends of the car, adjustable end slats, and the operating mechanism for said parts, substantially as described.

448,104. **OX-YOKE.** WILLIAM J. ACKERMAN, Malcolm, Wis. Filed Dec. 10, 1890. Serial No. 374,212. (No model.)

*Claim.*—1. A cast-metal yoke composed of a hollow stock having bow-openings, and having tabular extensions on the inner side of the stock in line with the bow-openings and extending flush with the top and the bottom sides of the stock, and having openings *g* in the top side opposite the neck-cavity, and the zinc plates fitted to the neck-cavities and having their ends bent over the sides of stock into the said openings *g*, substantially as described.

2. The combination, with the hollow cast-metal stock having bow-openings, and having portions cut from the edges of the top side at *g* opposite the neck-cavities, of the bows H and the zinc plates lining the said neck-cavities of the stock and having their ends bent over the edges of the said stock, substantially as described.

448,060. **HORSESHOEING-RACK.** SAMUEL M. MARTIN, Sidney, Ohio. Filed Aug. 14, 1890. Serial No. 362,026. (No model.)

*Claim.*—In the device herein shown and described, the combination, with a scaffold, of a supporting-rack suspended from the said scaffold and adapted to retain the animal, a transversely-shifting bar arranged at the top of the scaffold and to the rear of the rack, a pulley attached to said bar, and a rope passing over said pulley, adapted to be attached at one end to the hind leg of the animal, substantially as and for the purpose described.

## Figures and Tables

**Figure f1:**
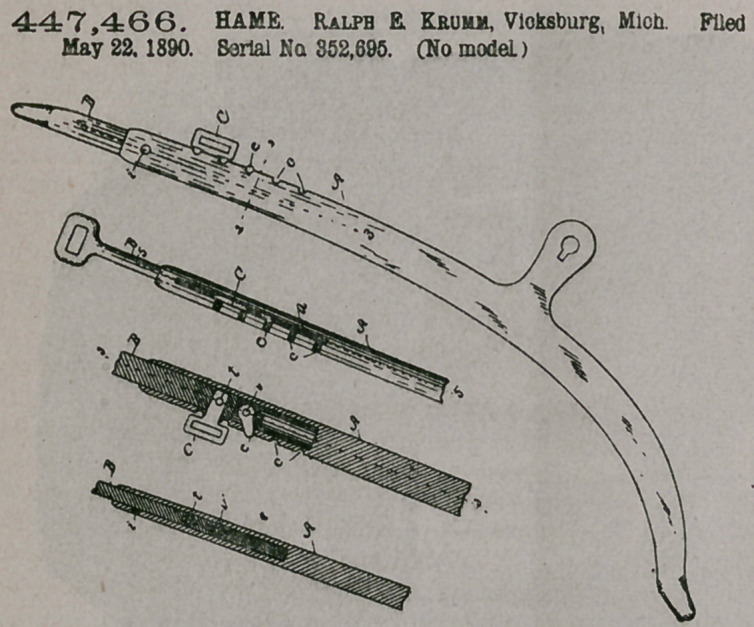


**Figure f2:**
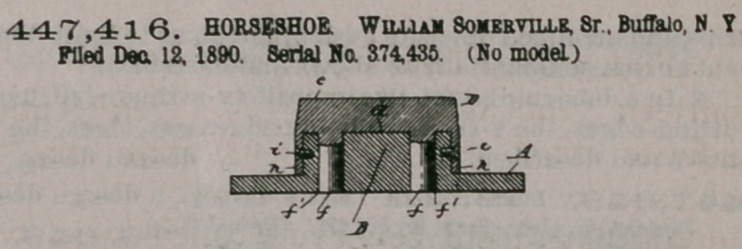


**Figure f3:**
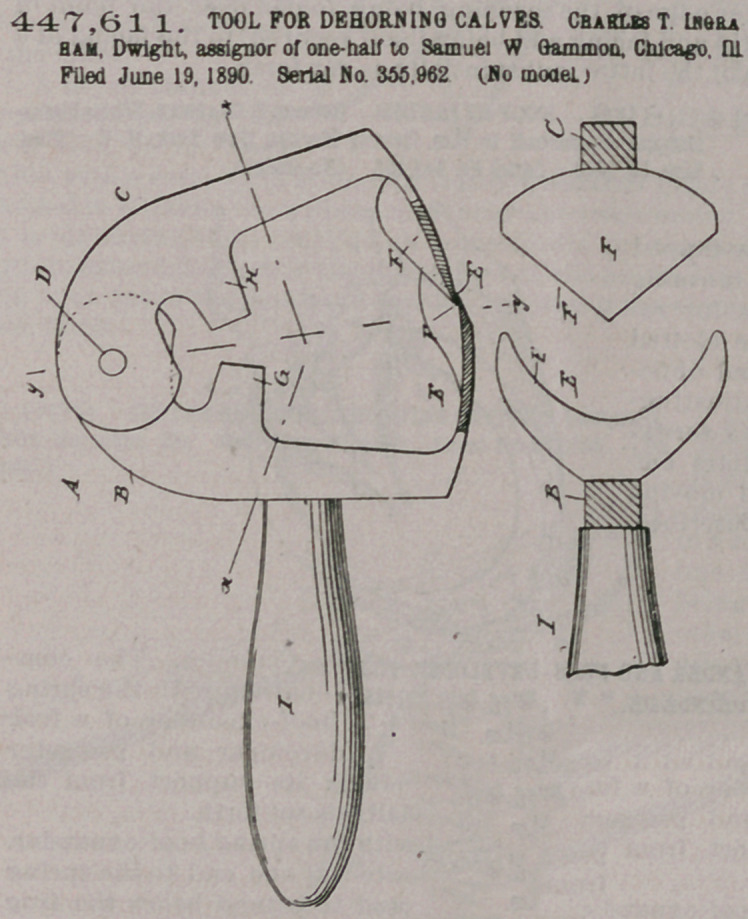


**Figure f4:**
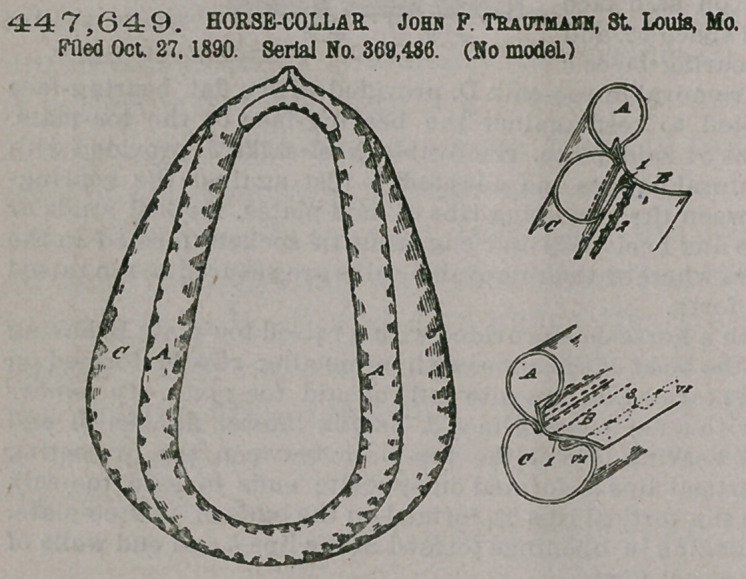


**Figure f5:**
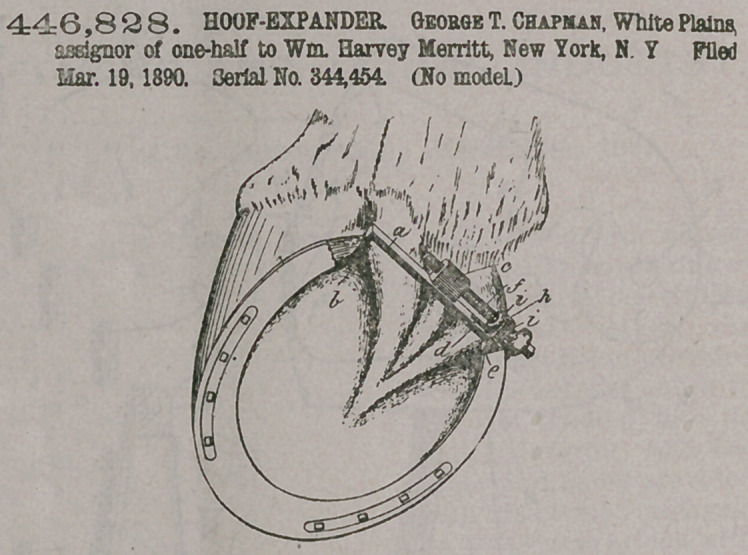


**Figure f6:**
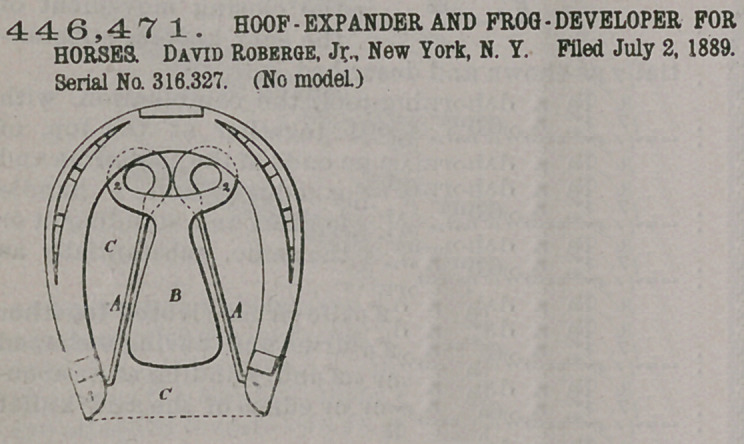


**Figure f7:**
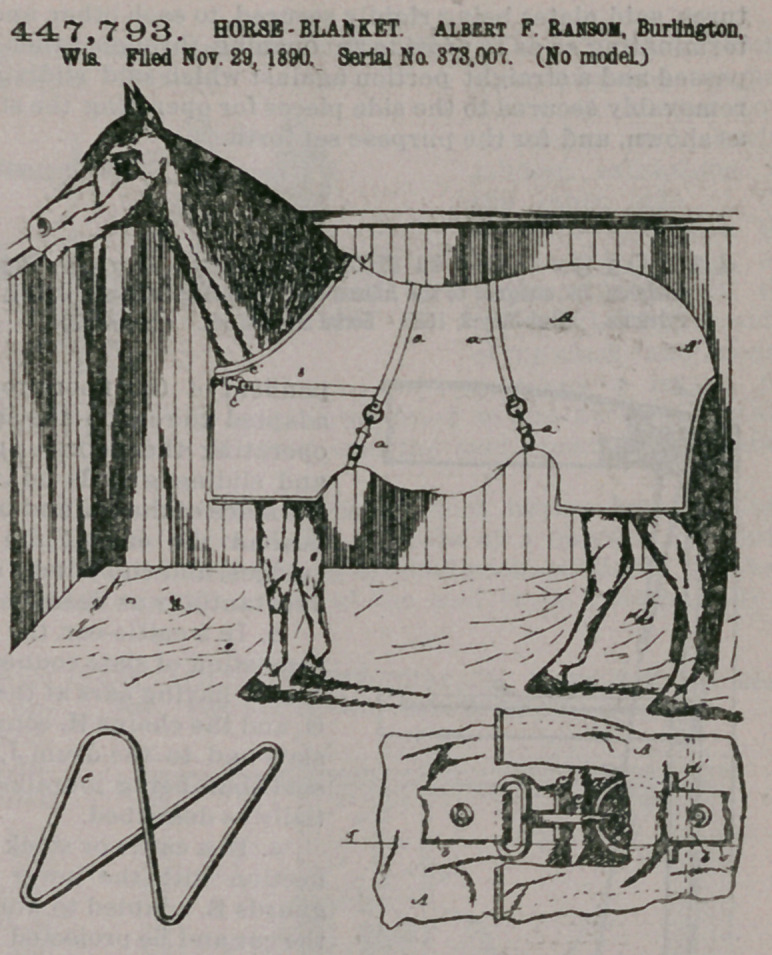


**Figure f8:**
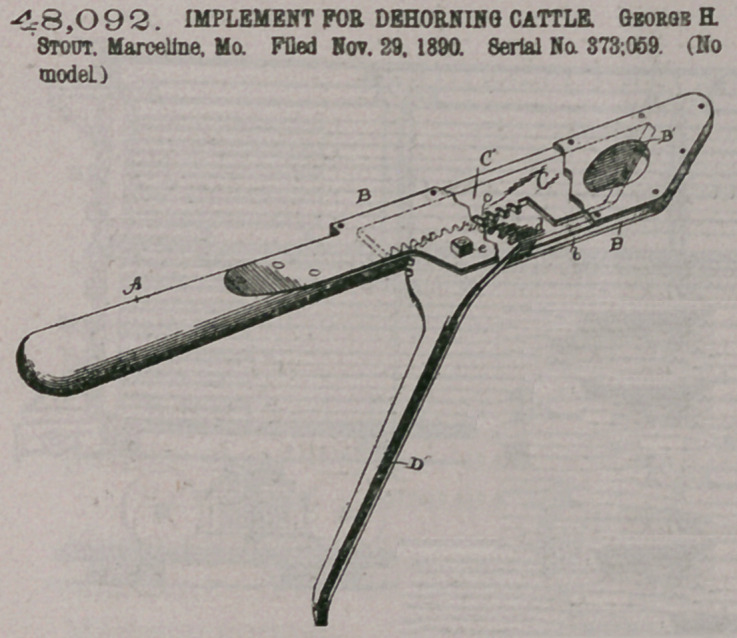


**Figure f9:**
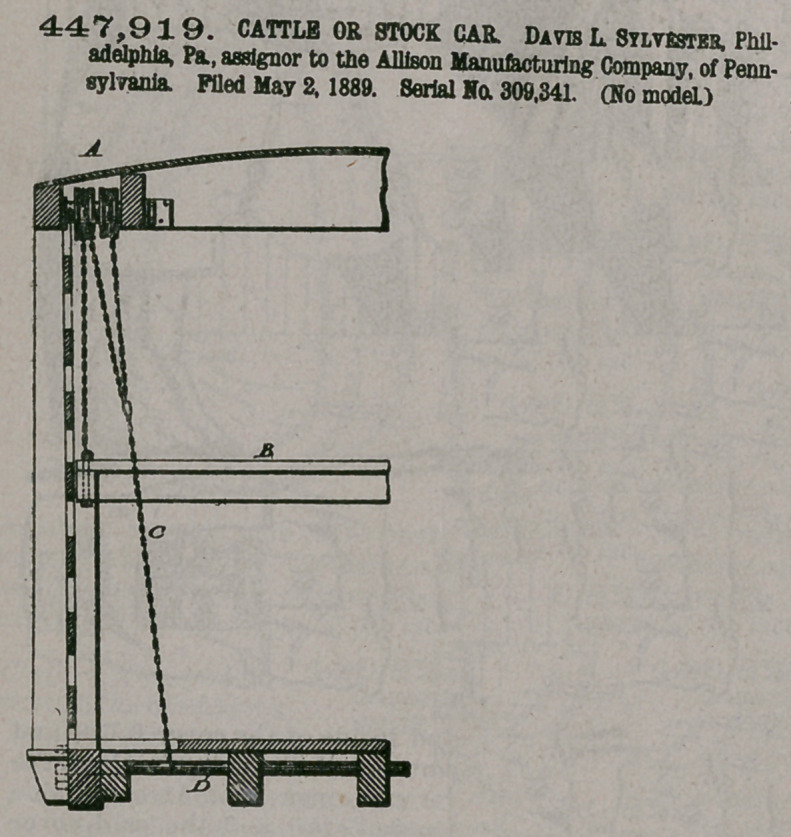


**Figure f10:**
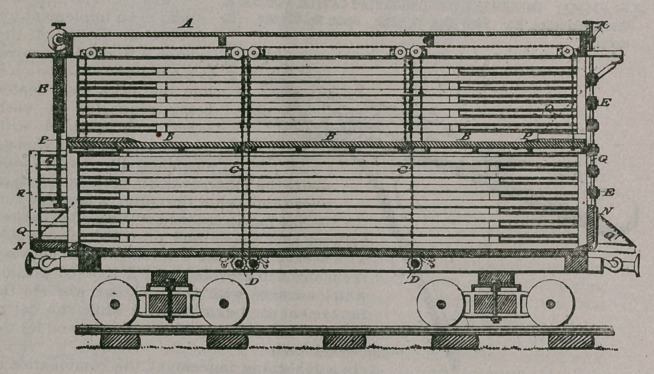


**Figure f11:**
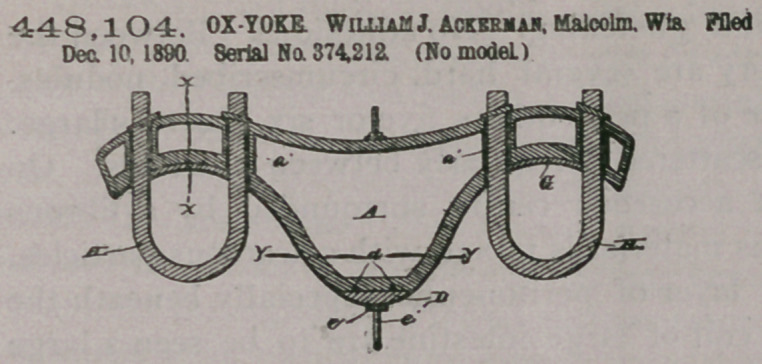


**Figure f12:**